# Higher Adolescent Body Mass Index Is Associated with Lower Regional Gray and White Matter Volumes and Lower Levels of Positive Emotionality

**DOI:** 10.3389/fnins.2016.00413

**Published:** 2016-09-08

**Authors:** James T. Kennedy, Paul F. Collins, Monica Luciana

**Affiliations:** ^1^Department of Psychology, University of MinnesotaMinneapolis, MN, USA; ^2^Center for Neurobehavioral Development, University of MinnesotaMinneapolis, MN, USA

**Keywords:** obesity, BMI, voxel based morphometry, adolescence, personality

## Abstract

Adolescent obesity is associated with an increased chance of developing serious health risks later in life. Identifying the neurobiological and personality factors related to increases in adiposity is important to understanding what drives maladaptive consummatory and exercise behaviors that result in obesity. Previous research has largely focused on adults with few findings published on interactions among adiposity, brain structure, and personality. In this study, Voxel Based Morphometry (VBM) was used to identify associations between gray and white matter volumes and increasing adiposity, as measured by Body Mass Index percentile (BMI%), in 137 adolescents (age range: 9–20 years, BMI% range: 5.16–99.56). Variations in gray and white matter volume and BMI% were then linked to individual differences in personality measures from the Multidimensional Personality Questionnaire (MPQ). After controlling for age and other covariates, BMI% correlated negatively with gray matter volume in the bilateral caudate (right: partial *r* = −0.338, left: *r* = −0.404), medial prefrontal cortex (partial *r* = −0.339), anterior cingulate (partial *r* = −0.312), bilateral frontal pole (right: partial *r* = −0.368, left: *r* = −0.316), and uncus (partial *r* = −0.475) as well as white matter volume bilaterally in the anterior limb of the internal capsule (right: partial *r* = −0.34, left: *r* = −0.386), extending to the left middle frontal subgyral white matter. Agentic Positive Emotionality (PEM-AG) was correlated negatively with BMI% (partial *r* = −0.384). PEM-AG was correlated positively with gray matter volume in the right uncus (partial *r* = 0.329). These results suggest that higher levels of adiposity in adolescents are associated with lower trait levels in reward-related personality domains, as well as structural variations in brain regions associated with reward processing, control, and sensory integration.

## Introduction

The high prevalence of obesity has become a major health concern. High body mass index (BMI, a measure of adiposity defined as weight (kg) divided by height (m^2^); Chumlea and Guo, 2000) raises the risks of developing a number of serious medical disorders, not limited to diabetes, cancer, and heart disease (Wyatt et al., 2006). Being overweight or obese during childhood and adolescence is associated with significant increases in later life morbidity for metabolic and cardiac diseases and with increased mortality (Reilly and Kelly, 2011). Using national norms, people are classified as overweight if they fall in the 85–95th weight percentile for age, sex, and height, and as obese if they fall in the 95th percentile or higher (Kuczmarski et al., 2002). Obesity rates vary by age, sex, and ethnicity (Ogden et al., 2014). Using the most recently published Center for Disease Control and Prevention (CDC) growth charts (from 2000) and conventional percentile cut-offs, obesity rates in American children and adolescents have risen over time to 20.5% with another 14% classified as overweight (Ogden et al., 2014). Understanding neurobiological correlates of increased adiposity and whether they relate to individual differences in personality traits linked to obesity is important in identifying mechanisms of weight gain and maintenance of unhealthy weight.

Previous research has examined how brain structure varies in relation to measures of body fat. A recent review found that higher adiposity was most commonly associated with lower gray matter volume, primarily in prefrontal and limbic regions in adolescents and younger adults, and spreading to the temporal and parietal lobes in older adults (Willette and Kapogiannis, 2015). The MRI findings in adolescents converge with behavioral studies that have linked higher adiposity with weaker executive functioning and inhibitory control, along with lower levels of academic achievement (Reinert et al., 2013; Liang et al., 2014). Together, these behavioral patterns suggest the possibility of compromised functioning in prefrontal and limbic-striatal regions that regulate interactions between higher-level cognitive control processes and appetitive motivational drives. Nevertheless, as detailed by Willette and Kapogiannis (2015), inconsistencies exist in both the adolescent and adult research literatures on structural MRI correlates of adiposity, and findings are especially mixed for measures of white matter.

The current study employed voxel-based morphometry (VBM) to investigate associations of BMI percentile (BMI%) and personality trait scores with gray and white matter tissue volumes in whole-brain T_1_-weighted MRI data. Previous VBM research in adolescents has found higher gray matter volume in the overweight and obese, relative to normal weight individuals, in the right hippocampus (functionally linked to increased motivation to eat and emotional eating; Wang et al., 2006) as well as lower gray matter volume in the left precentral gyrus (Moreno-Lopez et al., 2012). A study in children aged 8–10 found that greater adiposity was associated with lower gray matter volumes in the middle temporal gyrus, thalamus, superior parietal lobule, pre and postcentral gyri, and cerebellum (Ou et al., 2015). Yokum et al. (2012) studied adolescents and young adults [mean(SD) age 18(2.8) years], finding that body mass index (BMI) was positively correlated with gray matter volume in the middle occipital gyrus. Positive correlations between BMI and white matter volume were found in the middle occipital gyrus, middle temporal gyrus, fusiform gyrus, parahippocampal gyrus, rolandic operculum, and dorsal striatum, areas involved in food receipt cue processing and reward (Yokum et al., 2012). In the same study, lower prefrontal gray matter volumes predicted weight gain at a 1-year follow-up, though potential age effects were not controlled. These findings were interpreted as indicative of links between high BMI and poor inhibitory control and food related decision making, both of which were hypothesized to contribute to future weight gain (Yokum et al., 2012). One study found that obesity prone individuals had lower orbitofrontal gyrus, cerebellum, and insula gray matter volumes, suggesting that these regional variations precede obesity (Smucny et al., 2012).

Relevant findings also have been reported from studies using tissue volumes computed within *a priori* regions of interest (ROIs), as well as analyses of cortical thickness. Adolescent adiposity was correlated negatively with the volume of the frontal lobe, limbic system (hippocampus, parahippocampus, amygdala, cingulate, and cerebellum; Alosco et al., 2013), and orbitofrontal cortex, and both adiposity and orbitofrontal volume were correlated negatively with disinhibited eating behavior (Maayan et al., 2011). Examinations of cortical thickness in adolescents have suggested a negative relationship between adiposity and thickness in the orbitofrontal cortex (Yau et al., 2014; Ross et al., 2015; Sharkey et al., 2015).

Perhaps surprisingly, studies of overweight and obese adults have produced inconsistent results as well, both in terms of anatomical locations of effects and even their direction (Willette and Kapogiannis, 2015). For example, some adult VBM studies have reported higher gray matter volumes in the obese relative to normal weight individuals in the putamen, nucleus accumbens, cerebellum, orbitofrontal cortex, occipital lobe, and middle and inferior frontal gyri (Pannacciulli et al., 2006; Horstmann et al., 2011; Lou et al., 2014; Mole et al., 2016), while others have reported lower gray matter volumes in similar regions such as the putamen, globus pallidus, thalamus, amygdala, hippocampus, insula, temporal lobe, supramarginal gyrus, fusiform, pre and postcentral gyrus, and frontal lobe (Pannacciulli et al., 2006; Raji et al., 2010; Karlsson et al., 2013; Lou et al., 2014; Janowitz et al., 2015). Higher white matter volumes in obese relative to healthy weight adults were found in the brainstem, putamen, temporal lobe, and cerebellum (Pannacciulli et al., 2006; Haltia et al., 2007; Raji et al., 2010; Janowitz et al., 2015). The differences in white matter volume in the left temporal lobe were reduced following a 6 week diet intervention (Haltia et al., 2007).

Adult cortical thickness analyses have also found a negative relationship with adiposity in the orbitofrontal cortex, as well as the superior frontal gyrus, pre and postcentral gyri, inferior parietal gyrus, fusiform gyrus, and inferior and superior temporal gyri (Isaac et al., 2011; Marques-Iturria et al., 2013; Veit et al., 2014). Adult ROI analyses have found lower gray matter volumes in the obese relative to normal weight in the amygdala, cingulate, insula, and frontal, temporal, parietal, and occipital lobes (Bobb et al., 2014). Longitudinal analyses found that increases over time in BMI were associated with decreases in gray matter volumes in the temporal and occipital lobes, increases in gray matter volume in the hippocampus, and increases in white matter volumes in the internal capsule and frontal and parietal lobes (Bobb et al., 2014). Overall, perhaps the only consistent correlate of higher adiposity has been lower gray matter volume in prefrontal regions.

A recent meta-analysis of functional MRI studies indicated higher activity in the medial prefrontal cortex (MPFC), caudate, cerebellum, parahippocampus, thalamus, superior temporal gyrus, superior parietal lobule, amygdala, and hippocampus in obese relative to healthy-weight children, adolescents, and adults when viewing food cues. Lower activity was reliably observed in the insula and superior frontal gyrus. This pattern was interpreted as indicative of greater awareness and reward processing of food cues and decreased interoceptive awareness in obese relative to healthy weight individuals when viewing food cues (Kennedy and Dimitropoulos, 2014).

Behavioral anchors, including those related to major domains of personality, are useful in the interpretation of BMI-related neural variations given that the significance of higher vs. lower levels of gray or white matter volumes is unclear. Multiple studies, most of which have grouped individuals on the basis of weight status, have linked obesity to individual differences in personality traits. A recent systematic review that analyzed both group-based and correlational effects found that degree of adiposity positively correlates with extraversion and neuroticism in adults, specifically traits linked to impulsivity (as measured by the Karolinska Scales of Personality; Gerlach et al., 2015) and reward seeking. Degree of adiposity was negatively correlated with conscientiousness. Although direction of causation cannot be determined by these associations, high levels of neuroticism and impulsivity could lead to increased emotional eating, less restrained eating, and eating when not hungry (Gerlach et al., 2015). Extraversion appears to be correlated positively with craving and negatively with dieting behavior in obese women (Gerlach et al., 2015). Conscientiousness is linked to self-discipline and is self-reported to be higher when individuals are trying to lose weight (Gerlach et al., 2015). One study (Moreno-Lopez et al., 2012) examined adiposity, personality, and brain structure, finding that reward sensitivity and positive urgency were negatively related to gray matter volume in the somatosensory cortex in lean but not overweight or obese adolescents. However, they were unable to find a relationship between personality and brain structure that directly related to BMI range. It remains unclear whether brain regions can be identified that are associated both with higher BMIs and these personality domains. However, there are multiple brain regions in which structural differences have been linked separately to obesity and to personality traits, e.g., the prefrontal cortex has been linked to inhibition and food related decision making in obesity (Kennedy and Dimitropoulos, 2014; Jensen and Kirwan, 2015) as well as to the trait dimension of Conscientiousness in the Five Factor Model of personality (DeYoung et al., 2010; Vainik et al., 2013).

While neuroimaging research has indicated that brain regions associated with decision making, inhibitory control, interoception, reward, and motivation may vary in their structure and function in the context of frank obesity, the relevance of these findings to the full range of BMI is unclear. Furthermore, the findings in general have been inconsistent, particularly in adult samples. There is relatively little research examining brain structural correlates of BMI in children and adolescents, and associations among personality traits, BMI, and brain structure have not been addressed. This study addresses these limitations by focusing on associations between BMI and brain structure, using VBM, across a wide adolescent age range. Further, it examines how self-reported personality traits relate to the observed associations. While adult VBM findings report both positive and negative relationships between adiposity and gray or white tissue volume, the adolescent literature more reliably suggests that BMI% is negatively associated with regional gray matter volumes (Maayan et al., 2011; Smucny et al., 2012; Alosco et al., 2013; Yau et al., 2014; Ou et al., 2015) and positively associated with regional white matter volumes (Yokum et al., 2012; Ou et al., 2015). Based on the extant literature (Moreno-Lopez et al., 2012; Yokum et al., 2012; Vainik et al., 2013; Gerlach et al., 2015), it was hypothesized that striatal, prefrontal, and anterior cingulate gray matter tissue volumes would negatively correlate with BMI. In contrast, striatal white matter (particularly in the region of the dorsal striatum) was expected to positively correlate with BMI. These correlations were expected to be at least partially independent of age effects on tissue volumes, representing BMI-related variations in brain structure independent of developmental effects. BMI was hypothesized to be positively related to personality measures related to reward seeking and negatively related to measures of inhibition and conscientiousness. Gray and white matter tissue volumes in the dorsal striatum, anterior cingulate, and medial prefrontal cortex, hypothesized to be associated with higher body mass were expected to positively correlate with personality measures related to reward seeking (e.g., positive correlations of striatal volumes with MPQ Positive Emotionality) and negatively with measures related to conscientiousness and inhibition (e.g., negative correlations of prefrontal volumes with MPQ Constraint). The age range of the current sample also permitted us to explore whether neural variations associated with BMI% varied by participant age and whether observed age effects were linear vs. non-linear in nature. Accordingly, interactions between age and BMI% were modeled to explore this possibility.

## Materials and methods

### Participants

Participants were drawn from a longitudinal study of adolescent brain and behavioral development (Olson et al., 2009; Luciana et al., 2009; Urosevic et al., 2012). Informed consent was obtained from parents and assent from participants under the age of 18. Individuals aged 18 and older provided informed consent. Healthy participants, ages 9–23 (*n* = 203), were recruited between 2004 and 2006 from a community database of research volunteers maintained by the Institute of Child Development at the University of Minnesota, by postcard mailings to non-academic employees of the University, and by flyers posted throughout the university campus. Potential participants were excluded if they had been diagnosed with a psychological or neurological disorder, had chronic physical illnesses, were born preterm or had other birth complications, were non-native English speakers, used psychoactive substances, had uncorrected vision or hearing difficulties, were non-right handed, or if they could not be safely scanned or presented with contraindications to scanning (i.e., had metallic implants, full orthodontic braces, etc.). The protocol was approved by the Medical/Biological Committee of the University of Minnesota's Institutional Review Board.

The BMI percentile is only available from normative data for individuals under the age of 20. Similar percentile-based norms do not exist for adult samples. Thus, for the current analyses, only potential participants within the age range where child and adolescent BMI percentile (BMI%) could be calculated (e.g., 9–20 year-olds; Kuczmarski et al., 2002) and who were not underweight (< 5th BMI percentile) were included. This restriction reduced the sample to 140 participants. Three additional participants were excluded from the VBM analyses due to poor warping (see below), leaving a total of 137 participants (age 9.3–19.7 years, BMI% range 5.2–99.6th, *n* = 68 female) for the VBM analyses. See Table 1 for more information.

**Table 1:** **Demographics**.

**VBM Sample**					**Combined VBM and MPQ Sample**				
**Sex**	**Male**	**Female**	**Total**	**M v**.	**Sex**	**Male**	**Female**	**Total**	**M v**.
**N**	**69**	**68**	**137**	**F p**	**N**	**60**	**53**	**113**	**F p**
Age	14.88 (2.92)	14.89 (3.28)	14.89 (3.1)	NS	Age	15.58 (2.44)	16.17 (2.259)	15.86 (2.37)	NS
BMI%	58.62 (28.73)	60.12 (25.03)	59.36 (26.87)	NS	BMI%	57.13 (29.85)	60.09 (23.69)	58.52 (27.06)	NS

*Demographics for the VBM and VBM+MPQ analyses. VBM, Voxel based morphometry; MPQ, Multidimensional Personality Questionnaire; BMI%, Body Mass Index Percentile; M v. F p, Significance of gender difference in demographics; NS, Non-significant*.

### Measures

#### BMI percentiles

Height and weight were directly measured using a standard analog scale in a clinical research setting and converted to BMI% using the 2000 CDC growth charts (Kuczmarski et al., 2002). These charts contain the most recently collated norms for the studied age range.

#### MRI acquisition and preprocessing

Three-dimensional T_1_ weighted images were obtained from each participant using a Magnetization Prepared Rapid Gradient Echo (MPRAGE) scan (coronal acquisition using an eight-channel head coil array, *TR* = 2530 ms, *TE* = 3.65 ms, *TI* = 1100 ms, 240 slices, no gap, voxel size 1 mm^3^, flip angle = 7°, *FOV* = 256 mm) on a 3T Siemens Trio Scanner (Siemens Medical Systems, Erlangen, Germany) at the Center for Magnetic Resonance Research at the University of Minnesota. DICOM files were converted to nifti volumes (as required by SPM) using the mri_convert function of FreeSurfer (http://surfer.nmr.mgh.harvard.edu/fswiki). Statistical Parametric Mapping 12 (SPM12) (Wellcome Department of Imaging Neuroscience, London, UK) was used to manually align T1 weighted images along the AC/PC plane then to segment the images into gray matter, white matter, and cerebral spinal fluid. Whole-brain gray and white matter volumes were established using the Diffeomorphic Anatomical Registration Through Exponentiated Lie Algebra (DARTEL) module. This module warps the previously segmented tissue classes into MNI space while preserving specific tissue volumes (Ashburner, 2007). Three participants were lost in this step due to failed warping. An 8 mm full width at half maximum Gaussian kernel was used to smooth the gray and white matter maps. Gray and white matter masks were created by taking the average of all individuals' respective tissue maps and thresholding them at 0.3 to create a binary mask (Ashburner, 2007). The JHU ICBM-DTI-81 white matter label atlas (Hua et al., 2008) was binarized and subtracted from the gray matter mask to ensure proper separation of gray vs. white matter in the basal ganglia. Manual edits were made to the gray matter mask to ensure proper hemispheric and gray and white matter tissue separation.

#### Multidimensional personality questionnaire

Self-reported personality traits were assessed using the Multidimensional Personality Questionnaire–Brief Form (MPQ-BF), which consists of 155 true/false questions (Patrick et al., 2002). The MPQ-BF was administered to study participants aged 11 and older, because item content may not be appropriate for younger individuals (Schissel et al., 2011). A total of 113 participants had both VBM and MPQ-BF data.

The MPQ-BF includes 11 primary scales and can be scored according to a three- or five-factor model of higher-order traits. The conventional three-factor model includes the higher-order traits of Positive Emotionality (PEM), Negative Emotionality (NEM), and Constraint (CON). PEM is derived from the primary scales of Wellbeing, Social Potency, Achievement, and Social Closeness. The five-factor model subdivides PEM into Agentic (PEM-AG: derived from Social Potency, Achievement, and Wellbeing) and Communal (PEM-CO: derived from Social Closeness and Wellbeing) factors (Tellegen and Waller, 2008). Extraversion, particularly aspects related to agency and approach to reward, has been linked to PEM-AG (Depue and Collins, 1999; Tellegen and Waller, 2008). In the three-factor model, NEM is derived from the primary scales of Alienation, Aggression, and Stress Reaction. On rational rather than factor-analytic grounds, Tellegen's five-factor model subdivides NEM into Agentic (NEM-AG; derived from Aggression and Stress Reaction) and Alienated (NEM-AL: derived from Alienation and Stress Reaction) factors, to provide counterparts to PEM-AG and PEM-CO. Constraint is derived from the primary scales of Control, Harm Avoidance and Traditionalism. Constraint is associated with Conscientiousness from the five-factor model of personality (McCrae and Costa, 1987; McCrae and John, 1992; Tellegen and Waller, 2008).

### Statistical approach

A three-step statistical approach was implemented after the examination of descriptive associations among variables. First, to establish the relationship between BMI% and tissue volume for the 137 available participants, separate multiple regressions of gray and white matter volume were performed on sex, age, BMI%, the interaction of BMI% and age, and the respective tissue classes' total volume within whole-brain VBM maps using SPM12. To rule out the possibility of interactions between BMI% and sex and to determine whether separate equations were required for males and females, we modeled BMI%-by-sex interactions in initial analyses. No significant findings emerged, so this term was omitted from subsequent analyses to preserve statistical power. Regression analyses were then run on the full sample. All results surviving a familywise error correction of.05 using Threshold Free Cluster Enhancement (TFCE; Smith and Nichols, 2009) are reported here (TFCE SPM toolbox version r77, Gaser, 2015). TFCE combines local cluster extent and peak voxel height to identify regions with significantly elevated statistical parameter values. The cluster threshold is set using permutation testing; 5000 permutations were used in this study. Regional volume values, extracted from nine 5 mm radius spheres centered on cluster peaks, were used for regression analyses in SPSS version 22 (see the third step below). Partial correlations (controlling for age and sex) were computed between BMI% and total volumes for gray and white matter, to check for any confounds due to associations with global tissue volume.

Second, to measure associations between personality traits and BMI%, raw scores for the five-factor MPQ model (PEM-AG, PEM-CO, NEM-AG, NEM-AL, and CON) were entered with age and sex into a multiple regression equation predicting BMI% for the 113 available participants.

Third, the personality factors that significantly predicted BMI% were used as the dependent variables in a multiple regression equation with age, sex, total tissue class volumes, and extracted gray and white matter volume values from each significant cluster (from step 1 above) as predictors.

## Results

### BMI% and demographic characteristics

BMI% (mean 59.36, standard deviation 26.87, range 5.16–99.56) was not significantly associated with age (*r* = −0.055, *p* = 0.524) and was not significantly different between sexes (*t* = −0.324, *p* = 0.746), confirming the lack of cohort effects in the data. As expected, there were significant age and gender associations with global gray matter volumes (age *r* = −0.299, *p* < 0.001, gender *t* = 6.288, *p* < 0.001, males greater than females). There were also significant effects for global white matter volume (age *r* = 0.245, *p* = 0.004; gender *t* = 7.514, *p* < 0.001, males greater than females). After controlling for age and sex, BMI% was not correlated with global brain gray (partial *r* = 0.02; *p* = 0.814) or white (partial *r* = −0.008; *p* = 0.922) matter volumes. Age and sex are controlled in all subsequent analyses. VBM analyses also control for global brain volumes.

### VBM associations with BMI percentiles

**Figure 1:**
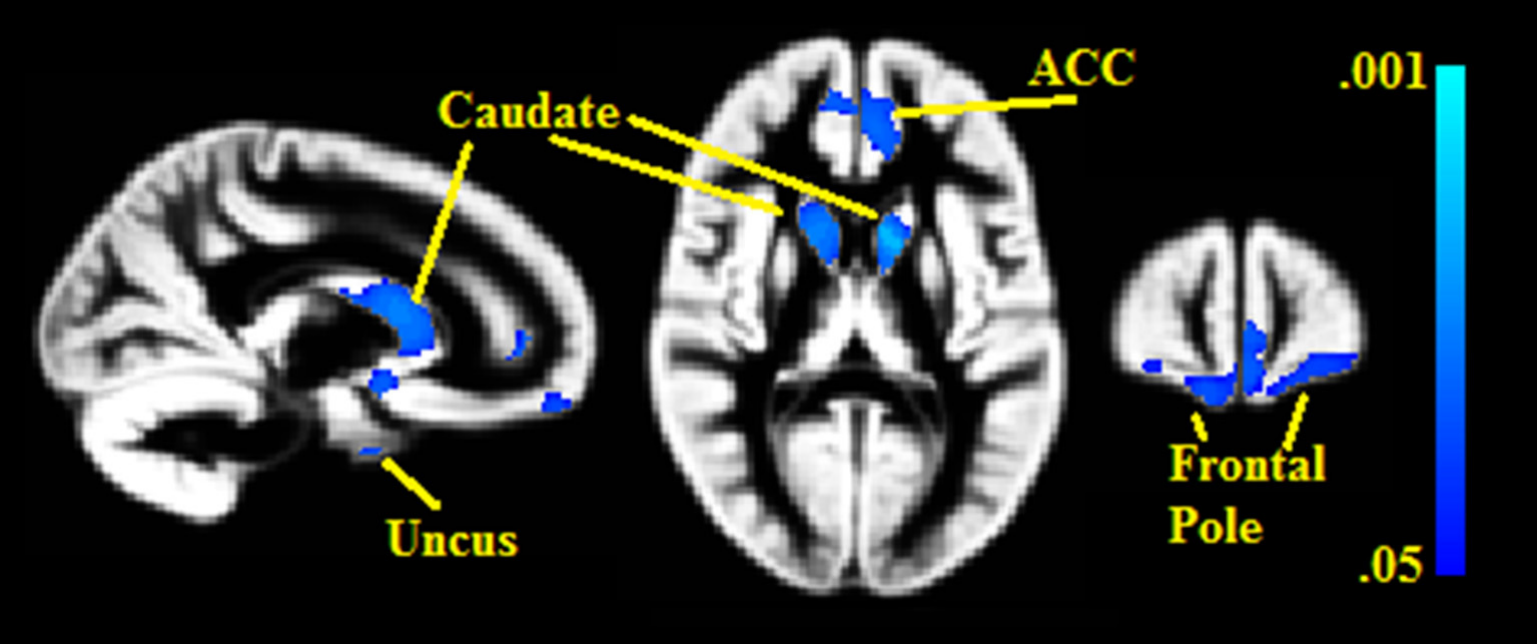
**Clusters where gray matter densities are negatively associated with BMI% shown in blue**. ACC, Anterior cingulate cortex. VBM clusters overlaid onto the gray matter DARTEL warp template. Centered on MNI Coordinates 13, 58, 11. FWE-corrected *p*-values shown from 0.001 (light blue) to 0.05 (dark blue).

Seven gray matter volume clusters were found to negatively correlate with BMI% (see Table 2 and Figure 1) after controlling for relevant covariates as described above. Clusters were found in the bilateral caudate (left pFWE = 0.002, right = 0.023), right uncus (pFWE = 0.003), left anterior cingulate, extending into the medial prefrontal cortex (pFWE = 0.014), bilateral frontal pole (left pFWE = 0.031, right = 0.044) and right medial prefrontal cortex (pFWE = 0.036). There were no statistically significant gray matter regions where volumes correlated positively with BMI% after correction for multiple comparisons.

**Table 2:** **Significant correlations between BMI percentile and gray and white matter volumes**.

**Peak area**	**Type**	**Vol**	**pFWE**	**Pr**	**H**	**MNI(X,Y,Z)**			**BA**
Caudate head	GM	6978	0.002	−0.404	L	−9	5	−2	–
		2432	0.023	−0.338	R	13	9	11	–
Anterior cingulate cortex		6943	0.014	−0.312	L	−6	41	4	11/32
Uncus		867	0.003	−0.475	R	14	3	−38	28/36
Frontal pole		853	0.031	−0.316	L	−21	60	15	11
		82	0.044	−0.368	R	10	57	−20	11
Medial prefrontal cortex		438	0.036	−0.339	R	2	44	31	32
Anterior limb of the internal capsule	WM	4883	0.008	−0.386	L	−18	13	13	–
		5440	0.014	−0.34	R	22	13	15	–

*GM, Gray Matter; WM, White Matter; H, Hemisphere; L, Left; R, Right; Vol, volume; pFWE, peak voxel TFCE significance at a Family Wise Error of 0.05. Pr, Partial correlation between BMI% and peak tissue volume (5 mm sphere), controlling for age, gender, BMI X age, and global tissue volume; MNI(XYZ), Montreal Neurological Institute coordinate; BA, Brodmann Area*.

**Figure 2:**
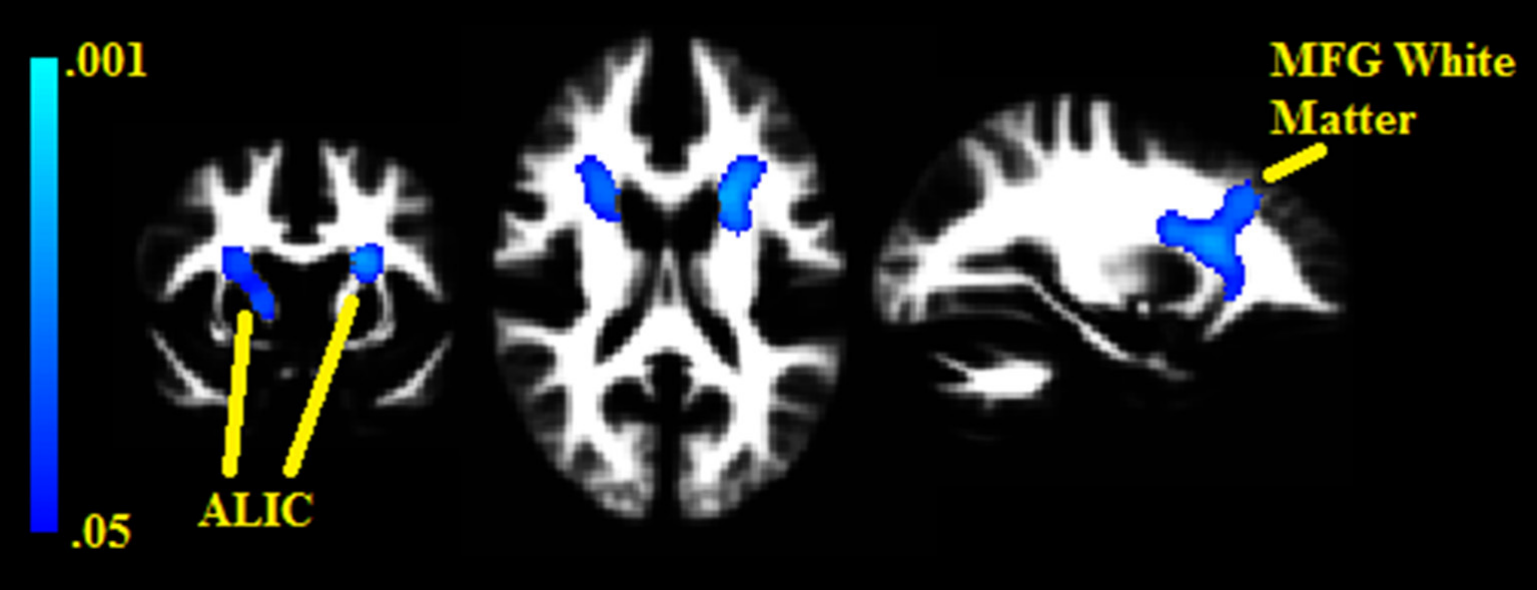
**Clusters where white matter densities are negatively associated with BMI% shown in blue**. ALIC, Anterior limb of the internal capsule. MFG, Middle frontal gyrus. VBM clusters overlaid onto the white matter DARTEL warp template. Centered on MNI Coordinates −24, 7, 17. FWE-corrected *p*-values shown from 0.001 (light blue) to 0.05 (dark blue).

Two white matter clusters, one in each hemisphere, were found lateral to the caudate (pFWE = 0.008 in the left hemisphere, pFWE = 0.014 in the right); each negatively correlated with BMI% (see Figure 2). These clusters represent portions of the anterior limb of the internal capsule, with the left hemisphere cluster extending into the middle frontal subgyral white matter. There were no white matter clusters that positively correlated with BMI%.

For both gray and white matter volumes, analyses examining the interactions between BMI% and age, BMI% and sex, and BMI%, age, and sex did not yield any significant findings. All BMI% and VBM volume relationships remained significant (*p* < 0.05) when restricted to the 113 participants with MPQ data. Uncorrected VBM results available in the supplementary information.

### Associations between BMI and self-reported personality traits

The five MPQ factors were examined for their association with BMI% using linear multiple regression analyses. Only PEM-AG significantly predicted BMI%. In a reduced model with the non-significant MPQ predictors removed, the overall adjusted *R*^2^ = 0.127; for PEM-AG as a predictor, *t* = −4.336, *p* < 0.001, partial *r* = −0.384, in an equation that also included age (partial *r* = 0.048) and sex (partial *r* = 0.138). Contrary to prediction, low PEM-AG was associated with higher BMI percentile scores.

**Figure 3:**
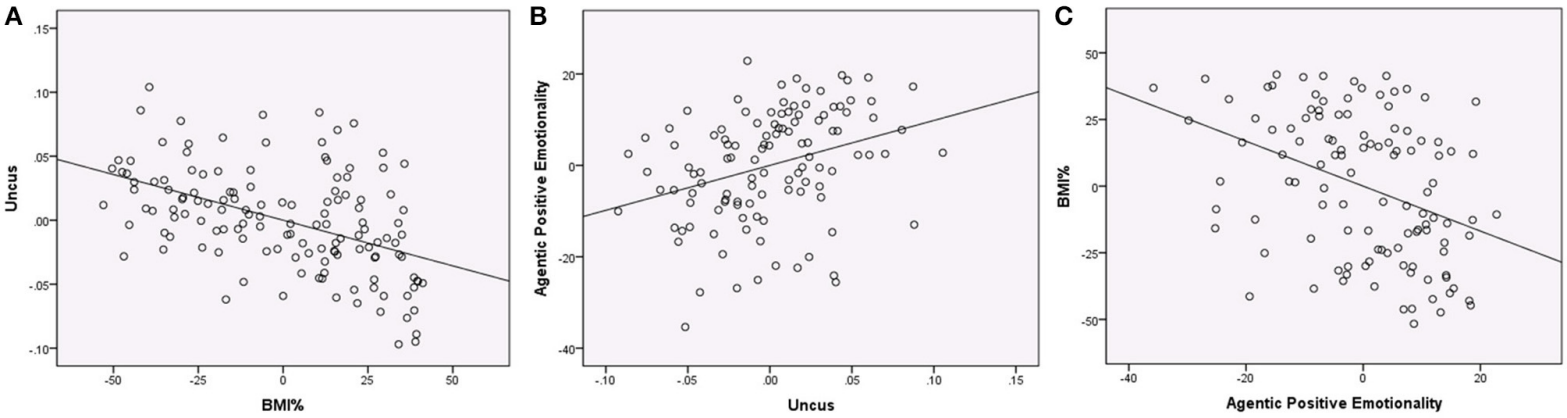
**Regression plots showing associations between BMI%, uncus gray matter volume, and Agentic Positive Emotionality (all variables centered)**. **(A)** Regression of BMI% on uncus gray matter volume (controlling age, sex, the interaction of BMI% and age, and global gray matter), partial *r* = −0.475, *p* < 0.001. **(B)** Regression of uncus gray matter volume on Agentic Positive Emotionality (controlling age, sex, and global gray matter), partial *r* = 0.320, *p* = 0.001. **(C)** Regression of Agentic Positive Emotionality on BMI% (controlling age and sex), partial *r* = −0.384, *p* < 0.001.

We next examined whether PEM-AG could be predicted from VBM data using the nine regions (7 gray matter and 2 white matter) that were significant in the VBM analyses. When predicting PEM-AG from VBM volumes that emerged as significant in the primary analysis (including age, sex, and global tissue volumes in the multiple regression equation), only the gray matter cluster in the right uncus emerged as a significant predictor. After removing the non-significant predictors, the reduced model had an *R*^2^ = 0.188 (adjusted *R*^2^ = 0.158, *F* = 6.253, *p* < 0.001) and the right uncus cluster variable had a partial *r* = 0.329 (*t* = 3.623, *p* < 0.001). No other regions were significantly associated with PEM-AG (see Figure 3).

## Discussion

The goals of these analyses were to identify gray and white matter volumes that varied with BMI percentile, to identify personality measures related to BMI percentile, and to identify how these personality measures and tissue volumes were related. Based on prior literature (Moreno-Lopez et al., 2012; Yokum et al., 2012; Vainik et al., 2013; Gerlach et al., 2015), it was hypothesized that BMI percentile would negatively correlate with gray matter tissue volume in the striatum, cingulate, and prefrontal cortex and positively correlate with white matter volume near the striatum, be positively related to measures of reward seeking, and negatively related to measures of conscientiousness and inhibition. It was further hypothesized that the tissue volumes and personality measures would be significantly related to one another and independently related to BMI percentile. These hypotheses were partially supported. Gray matter volume in the medial prefrontal cortex, frontal pole, anterior cingulate, caudate, and uncus and white matter volume in the anterior limb of the internal capsule were negatively associated with BMI percentile. BMI percentile was also negatively associated with PEM-AG. PEM-AG correlated positively with gray matter volume in the uncus but did not correlate with volumes in the other regions that were significantly associated with BMI percentile.

The interpretation of lower gray and white matter volumes in relation to increasing BMI percentiles is complicated by the adolescent age range that was targeted in this investigation. With increasing age, adolescents are expected to show reductions in cortical gray matter volume (perhaps indicative of regressive maturational processes such as synaptic pruning; Sowell et al., 2001) as well as increases in white matter volume (perhaps indicative of increasing myelination and expanding axonal diameter; Ladouceur et al., 2012). These patterns were indeed observed in relation to global gray and white matter tissue volumes. Importantly, BMI percentile (already age-normed) did not increase with age, indicating the absence of cohort effects, and BMI percentile was not correlated with total gray and white matter volumes independent of select regional associations. Given that age was controlled in the VBM analysis, normative age-related gray matter volume reductions and white matter volume increases are unlikely to account for the patterning that we observed. In other studies, reductions in both gray and white matter volume have been attributed to both lesioning and early neurodevelopmental disruption through either reduced neuroproliferation or excessive dendritic pruning (Bernasconi et al., 2004; Mueller et al., 2006; Appenzeller et al., 2007; Chua et al., 2007; Inagaki et al., 2007; Mechtcheriakov et al., 2007). As this is a normative, non-clinical sample, significant volume reductions in the context of higher BMI% due to lesioning effects are highly unlikely. It is possible that the negative associations of BMI with gray and white matter volumes reflect genetic influences on early brain development that increase vulnerability to later obesity. For example, variation in the fat mass and obesity-associated gene (FTO) has been linked both to higher body fat mass and lower volumes of brain tissue, including both gray and white matter, possibly by influencing the allocation of stem cells toward brain cell lineages vs. adipose tissue lineages (Melka et al., 2013). This view suggests that the observed associations of lower gray and white matter volumes with higher BMI% develop very early in life and contribute to weight gain through alterations of brain circuits that regulate eating behavior. This formulation coheres with the conclusions of a recent large-scale epidemiological study that found similar patterns of lower gray matter using VBM (Janowitz et al., 2015).

Lower gray matter volumes were found in the medial prefrontal cortex, frontal pole, anterior cingulate, uncus, and caudate as BMI percentile increased, along with lower white matter volume in a region extending from the anterior limb of the internal capsule into the medial frontal gyrus. Most of these regions are associated with the anteromedial reward network, which features prominently in the study of both addiction and obesity (Volkow et al., 2013). Basal ganglia-thalamocortical circuits form the core of this network and are composed of anterior cortical regions, thalamus, and basal ganglia structures, along with their white matter projections (Alexander et al., 1986; Depue and Collins, 1999; Ikemoto et al., 2015). These circuits mediate both short- and long-term responding to reward cues (Volkow and Baler, 2015). With regard to reward structures involved in the current results, the anterior region of the caudate integrates phasic dopamine signals in the context of reward learning and responding to novelty (Guitart-Masip et al., 2010; Hikosaka et al., 2014; Volkow and Baler, 2015; Schultz, 2016), and a variety of studies have linked anterior caudate activity to eating behavior. For example, fMRI research has found the caudate to be more active in the obese relative to healthy-weight individuals when viewing food cues (Kennedy and Dimitropoulos, 2014), and this relationship is reversed when consuming rewarding food (although this is mediated by genetics; Stice et al., 2008). The white matter clusters were immediately adjacent to the gray matter caudate clusters and primarily overlapped the anterior limb of the internal capsule. This white matter is part of the core of the anatomical connectivity within the basal ganglia-thalamocortical circuits, including striatocortical and thalamocortical projections, e.g., to anterior cingulate, medial prefrontal, and frontal polar cortex, as well as subcortical connectivity from the caudate to the globus pallidus and thalamus (Kotz et al., 2013). In the left hemisphere the cluster covered white matter connecting the head of the caudate to the middle frontal gyrus, a region involved in decision making regarding risk and reward (Rushworth, 2008); functional activity within this region has been shown to vary in obesity (Kennedy and Dimitropoulos, 2014). Projections from the ALIC also terminate in the anterior cingulate and frontal pole (Kotz et al., 2013; Heilbronner and Haber, 2014), regions that had lower gray matter volumes as BMI% increased. The anterior cingulate is involved in reward motivation and mediation of response conflict, and has been specifically linked to disinhibition when viewing food stimuli (Kringelbach, 2004; Berthoud and Morrison, 2008; Martin et al., 2010). Adult obesity research has found lower cortical thickness and gray matter density in the frontal pole (Kurth et al., 2013; Marques-Iturria et al., 2013). The medial frontal pole (where the BMI%-gray matter volume correlation was strongest) has been linked to understanding of mental and emotional states (Gilbert et al., 2006). Difficulty understanding emotion has been linked to eating disorders (Bydlowski et al., 2005) and overeating of high calorie foods has been associated with emotional regulation (Macht, 2008).

Overall, the VBM results highlight the reward network and indicate that functional associations likely involve core reward-related processes: encoding of reward value, integrating reward predictions with outcomes (reward learning), mediating conflicting reward responses, decision making in regard to reward vs. risk and immediate vs. delayed reward, and self-regulation of emotion in the context of reward. We have addressed the prominence of reward-related processes in adolescent brain and behavioral development previously (Luciana et al., 2012; Luciana and Collins, 2012; Silverman et al., 2016), and the current study potentially extends this research into the domain of food reward and eating behavior. However, the negative association of uncus volume with BMI% is less easily incorporated into an interpretive framework based on the reward network and associated behavioral processes, given the limited research regarding the uncus' primary functions. The uncus lies in the temporal lobe, medial to the anterior tip of the parahippocampal gyrus and below the amygdala. However, despite its proximity to these key structures in emotion and memory, the uncus is part of the primary olfactory cortex and receives its input from the olfactory tract along the ventral surface of the frontal lobe. It is known in clinical neurology primarily for its association with partial complex seizures featuring strong unpleasant odors and/or tastes during the prodrome (Kiernan, 2012). In functional MRI (fMRI) studies of affective stimulus processing, the uncus appears to be involved preferentially in processing of aversive emotional stimuli (e.g., Beraha et al., 2012). Interestingly, fMRI research has found the uncus to be more active in anorexic participants relative to healthy controls when viewing images of their own bodies, but not when processing other negative emotional stimuli (Zhu et al., 2012). However, structural MRI research has not, to our knowledge, linked the uncus to body mass, adiposity, or eating behavior. Our observed correlation of lower uncus gray matter volume with higher BMI might reflect the influence of olfactory processing on eating behavior, given that olfactory and gustatory flavor signals integrate automatically and relatively early in flavor processing (Veldhuizen et al., 2010).

Additionally, regression analyses found that gray matter volume in the right uncus was positively related to PEM-AG, which in turn was negatively related to BMI percentile. These results are surprising as adult personality studies have positively linked reward seeking to adiposity (Gerlach et al., 2015) and PEM-AG is positively linked to reward seeking (Depue and Collins, 1999). The mutual associations observed among these three variables are intriguing and add a novel nuance to the current literature. As noted above, the uncus is part of the primary olfactory cortex and increased uncus activation appears to be linked to processing of aversive stimuli; hence, greater gray matter volume in the uncus might be expected to result in higher negative emotionality, rather than positive emotionality. However, the uncus also represents a point of origin for the uncinate fasciculus, a white matter fiber tract connecting the anterior temporal lobe to the orbitofrontal cortex that facilitates higher-order cognitive control over emotional behavior (Von Der Heide et al., 2013), e.g., the persistence of long-term goal pursuit despite frustrating obstacles. This form of behavior is measured by PEM-AG and especially its primary subscale, Achievement, and reflects a personality domain that may be connected to existing reports of weaker cognitive inhibitory control and lower academic achievement in adolescents with higher adiposity levels (Reinert et al., 2013; Liang et al., 2014). Thus, if the uncus finding is a marker of connectivity rather than local integration, its significance in terms of eating behavior lies in weaker temporal-frontal interactions in the control of long-term reward behavior, such as delay of gratification, rather than in weaker olfactory-gustatory integration. These interpretations are admittedly speculative. Additional research is required to resolve this basic question regarding functional significance.

While the negative associations of BMI% with regional gray matter volumes are consistent with prevailing trends in adolescent adiposity research, previous VBM research often has found a positive relationship between adiposity and white matter volume, particularly in the striatal region (Pannacciulli et al., 2006; Yokum et al., 2012), though the overall relationship between adiposity and white matter volume remains unclear (Willette and Kapogiannis, 2015). It appears that inconsistencies between previous research and the results presented here may be due to differences in data analysis and sample composition. In the Yokum et al. (2012) paper, the striatal white matter findings are within gray matter ROI masks in very small clusters (totaling 14 voxels) rather than within the adjacent white matter tracts where our findings occur. While the cluster found in the Pannacciulli et al. (2006) paper were more extensive (561 voxels), it appears to localize to the right external capsule ventral to the putamen whereas ours occur in the bilateral internal capsule lateral to the caudate. The Pannacciulli et al. (2006) findings occur in an adult sample. Therefore, it may be that the relationship between obesity and striatal white matter varies in direction and location at least partly due to age. The lack of temporal and parietal findings noted in adult research may reflect age related differences observed primarily in middle aged and older adults (Willette and Kapogiannis, 2015). The current study included a BMI% by age interaction in the regression model to account for any potential interaction between adiposity and brain development. The lack of any significant finding suggests that if brain developmental patterns vary by BMI%, such variations are occurring outside of the range of adolescence. Across studies, there are also differences in the samples' BMI distributions. Other studies specifically recruited for obesity and had high numbers of frankly obese participants.

### Limitations

This study is not without other limitations. The original aim of this project was to study adolescent brain and psychological development. Consequently, although the sample varies in BMI status, rates of extreme obesity are relatively low. While BMI and BMI percentile are commonly used to reflect adiposity (e.g., Raji et al., 2010; Moreno-Lopez et al., 2012; Yokum et al., 2012; Alosco et al., 2013; Karlsson et al., 2013; Bobb et al., 2014; Marques-Iturria et al., 2015; Ou et al., 2015), they are poor in distinguishing whether variance in weight is due to excess fat or to excess muscle [although both measures correlate very highly using measurement techniques that reliably differentiate between the two (*r* = 0.85 in children and adolescents); Steinberger et al., 2005]. There is some evidence that increased non-adipose tissue accounts for BMI-related differences in brain structure (Weise et al., 2013). Additionally, BMI-brain correlates were assessed using whole-brain VBM, which has less sensitivity than an analysis based on a limited set of well-chosen *a priori* ROIs, as informed by prior research. This type of analysis will be possible in future research if VBM studies begin to converge more consistently on specific neural structures involved in body weight maintenance during adolescence. The lack of MPQ-BF data for the youngest participants in the study limited the age range that could be examined in personality-BMI associations. Finally, eating behaviors, attitudes toward food, and body esteem were not measured, so our interpretations regarding the functional significance of the observed associations in relation to such behaviors are speculative.

## Conclusions

The results of this study demonstrate that structural variations in regional brain volumes are associated with individual differences in body mass index in children and adolescents, and these variations can be further linked to individual differences in agentic positive emotionality. With increasing BMI%, we found lower gray matter volumes in the medial prefrontal, frontal pole, anterior cingulate, and caudate, and lower volume in the internal capsule and in striato-medial prefrontal white matter. Together, these results indicate that increasing BMI is associated with structural variation across the anteriomedial reward system, and suggest that reward-related behavior and reward vs. risk decision-making processes may also vary as BMI progresses from typical to slightly elevated to extreme levels associated with obesity. This dimensional patterning is an important future direction to be explored more fully. The findings for the uncus and Agentic Positive Emotionality suggest an as-yet unexplored mechanism involving emotional reactivity that interacts with both bodyweight levels and positive motivational drive toward long-term goals. Overall, the results in this adolescent sample are more likely to reflect neuroanatomical predictors rather than consequences of body weight levels, but longitudinal data will be required to resolve this issue. Future longitudinal research also will be necessary to build on the current BMI-based results and clarify the specific links between structural modulation of the reward network and long-term patterns of eating behavior.

## Author contributions

JK, PC, and ML all contributed to the design of the analysis and the writing of the manuscript.

### Conflict of interest statement

The authors declare that the research was conducted in the absence of any commercial or financial relationships that could be construed as a potential conflict of interest.
